# Macrolide Resistance and *In Vitro* Potentiation by Peptidomimetics in Porcine Clinical Escherichia coli

**DOI:** 10.1128/msphere.00402-22

**Published:** 2022-09-26

**Authors:** Yibing Ma, Mattia Pirolo, Prabha Subramani, Ronette Gehring, Peter Damborg, Henrik Franzyk, Luca Guardabassi

**Affiliations:** a Department of Veterinary and Animal Sciences, University of Copenhagen, Copenhagen, Denmark; b Institute of Risk Assessment Sciences, Utrecht University, Utrecht, The Netherlands; c Department of Drug Design and Pharmacology, University of Copenhagen, Copenhagen, Denmark; University of Nebraska Medical Center

**Keywords:** macrolide resistance, *E. coli*, pigs, enteritis, peptidomimetics, antibiotic potentiation

## Abstract

Escherichia coli is intrinsically resistant to macrolides due to outer membrane impermeability, but may also acquire macrolide resistance genes by horizontal transfer. We evaluated the prevalence and types of acquired macrolide resistance determinants in pig clinical E. coli, and we assessed the ability of peptidomimetics to potentiate different macrolide subclasses against strains resistant to neomycin, a first-line antibiotic in the treatment of pig-enteric infections. The erythromycin MIC distribution was determined in 324 pig clinical E. coli isolates, and 62 neomycin-resistant isolates were further characterized by genome sequencing and MIC testing of azithromycin, spiramycin, tilmicosin, and tylosin. The impact on potency achieved by combining these macrolides with three selected peptidomimetic compounds was determined by checkerboard assays in six strains representing different genetic lineages and macrolide resistance gene profiles. Erythromycin MICs ranged from 16 to >1,024 μg/mL. Azithromycin showed the highest potency in wild-type strains (1 to 8 μg/mL), followed by erythromycin (16 to 128 μg/mL), tilmicosin (32 to 256 μg/mL), and spiramycin (128 to 256 μg/mL). Isolates with elevated MIC mainly carried *erm*(B), either alone or in combination with other acquired macrolide resistance genes, including *erm*(42), *mef*(C), *mph*(A), *mph*(B), and *mph*(G). All peptidomimetic-macrolide combinations exhibited synergy (fractional inhibitory concentration index [FICI] < 0.5) with a 4- to 32-fold decrease in the MICs of macrolides. Interestingly, the MICs of tilmicosin in wild-type strains were reduced to concentrations (4 to 16 μg/mL) that can be achieved in the pig intestinal tract after oral administration, indicating that peptidomimetics can potentially be employed for repurposing tilmicosin in the management of E. coli enteritis in pigs.

**IMPORTANCE** Acquired macrolide resistance is poorly studied in Escherichia coli because of intrinsic resistance and limited antimicrobial activity in Gram-negative bacteria. This study reveals new information on the prevalence and distribution of macrolide resistance determinants in a comprehensive collection of porcine clinical E. coli from Denmark. Our results contribute to understanding the correlation between genotypic and phenotypic macrolide resistance in E. coli. From a clinical standpoint, our study provides an initial proof of concept that peptidomimetics can resensitize E. coli to macrolide concentrations that may be achieved in the pig intestinal tract after oral administration. The latter result has implications for animal health and potential applications in veterinary antimicrobial drug development in view of the high rates of antimicrobial-resistant E. coli isolated from enteric infections in pigs and the lack of viable alternatives for treating these infections.

## INTRODUCTION

Enteric infections are responsible for most antimicrobial use in pig production (1), including high-priority clinically important antimicrobials (2, 3). Enterotoxigenic Escherichia coli (ETEC) is the most important cause of enteric disease and death in suckling and weaned pigs (4). Colistin sulfate and zinc oxide have been used for several decades as the first choices for the treatment and control of ETEC enteritis in weaned pigs. Recently, the use of these compounds was restricted or banned in the European Union and in other parts of the world to prevent selection and zoonotic transmission of plasmid-mediated colistin resistance and methicillin-resistant Staphylococcus aureus (MRSA), respectively, resulting in a dearth of effective drugs for managing ETEC infections. Resistance to alternative antimicrobial drugs, such as the aminoglycoside neomycin, is on the rise due to their increased usage (2). A review recently published by the European Food Safety Authority (EFSA) classified ETEC as the most important multidrug-resistant (MDR) bacteria in pig production, highlighting the potential consequences on animal health and welfare associated with antimicrobial resistance in this pig pathogen (5). Thus, innovative therapeutic strategies are required for controlling ETEC-associated diseases in pig production.

Novel antibiotics with activity against Gram-negative bacteria are unlikely to be approved for veterinary use, since there is an urgent need for this type of drugs in human medicine. A possible way to circumvent this bottleneck in veterinary drug discovery is to use helper compounds to potentiate antimicrobials that are already authorized for use in animals. Macrolides comprise a large and diverse family of protein synthesis inhibitors that are broadly used in finisher pigs for the treatment of enteritis caused by Brachyspira hyodysenteriae and Lawsonia intracellularis (6). The distribution properties of macrolides are, in principle, suitable for management of bacterial enteritis because they are poorly absorbed following oral administration, and thus concentrate in the intestinal tract (7, 8). However, E. coli and other Gram-negative pathogens are intrinsically resistant to macrolides due to the low permeability of their polar outer membrane (9, 10). Moreover, they may acquire additional resistance by a variety of mechanisms, including target site modification by *erm* methylases, enzymatic inactivation by *ere* esterases or *mph* phosphotransferases, and *mef* efflux pumps (11). Yet, the prevalence of acquired resistance is largely unknown in E. coli because macrolides are not routinely included in the antibiotic panels used for susceptibility testing of clinical isolates.

Peptidomimetics are short synthetic peptide analogues that often have a modified backbone (e.g., including one or more carbons along the peptide chain), which confers improved enzymatic stability and lower production cost than that of larger cationic antimicrobial peptides (12). In a previous study (13), we showed that submicromolar concentrations (0.25 to 0.5 μM) of peptidomimetics can induce susceptibility to azithromycin in human MDR E. coli and Klebsiella pneumoniae. Possible repurposing of macrolides for treatment of Gram-negative infections was further corroborated by a following study on both azithromycin and clarithromycin (14). Nevertheless, these studies did not include any of the 16-membered macrolides used in pig practice, such as tylosin, tilmicosin, and spiramycin, and the relationship between resistance phenotype and the presence of acquired macrolide resistance genes was not investigated.

In the present study, we determined the prevalence and types of acquired macrolide resistance in a comprehensive Danish collection of porcine E. coli of clinical origin. Neomycin-resistant isolates were further characterized by genome sequencing to determine the prevalence and types of acquired macrolide resistance genes, and their relationship with phenotype. Six strains representing distinct E. coli lineages and resistance phenotypes were tested by checkerboard assays to assess possible synergistic interactions of three selected peptidomimetics with different subclasses of macrolides. This part of the study focused on neomycin-resistant strains because infections caused by strains resistant to this first-line antibiotic require innovative treatment strategies to overcome their multidrug resistance profiles.

## RESULTS

### Macrolide MIC distributions and resistance genotypes.

**FIG 1:**
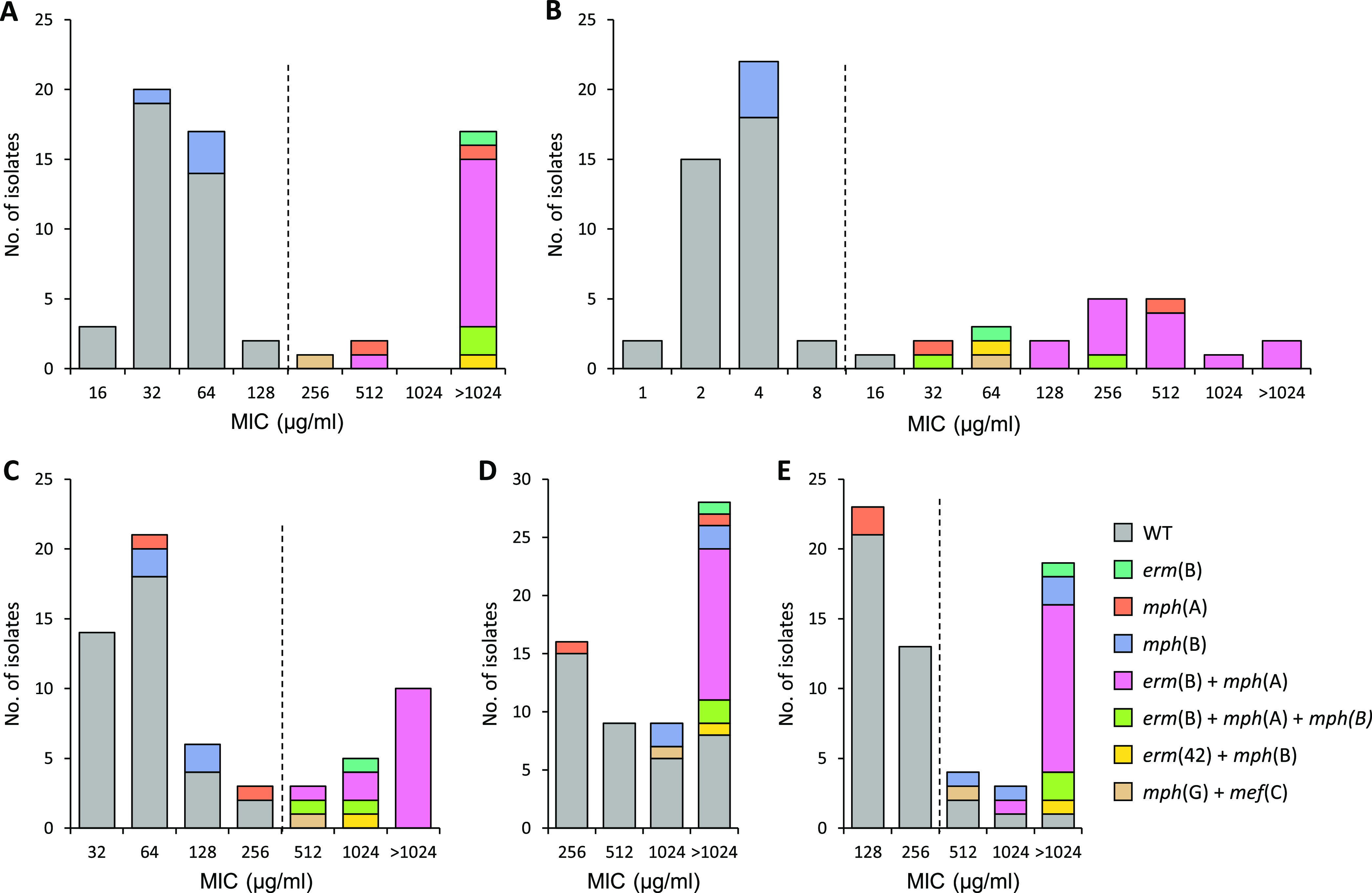
Distribution of macrolide resistance genes and MICs of erythromycin (A), azithromycin (B), tilmicosin (C), tylosin (D), and spiramycin (E) in 62 genome-sequenced MDR E. coli isolates from pigs in Denmark. The dashed line indicates the tentative epidemiological cutoff determined by ECOFFinder. WT, wild-type population.

The MICs of erythromycin ranged from ≤32 to >1,024 μg/mL (Table 1), with a clear bimodal distribution where highly resistant isolates displayed MIC values >1,024 μg/mL, accounting for 19.1% of the 324 isolates tested. Limited to 23 strains that showed erythromycin MICs of ≤32 μg/mL, the exact MIC values were further determined as 16 μg/mL for 3 strains and 32 μg/mL for the remaining 20, as shown in Fig. 1A. Sixty-two neomycin-resistant isolates were selected based on the EUCAST epidemiological cutoff (ECOFF) and presence of acquired resistance determinants. Wild-type strains that did not contain any macrolide resistance determinant had MICs between 16 and 128 μg/mL, and a tentative ECOFF value of erythromycin for clinical E. coli isolates was set at 128 μg/mL by using ECOFFinder (Fig. 1A). Acquired resistance was mainly associated with *mph*(A) (27.4%), either alone or in combination with other resistance genes, followed by *erm*(B) (25.8%), *mph*(B) (11.3%), *erm*(42) (1.6%), *mph*(G) (1.6%), and *mef*(C) (1.6%). Of the 16 *erm*(B)-positive isolates, 13 additionally contained *mph*(A), while 2 contained both *mph*(A) and *mph*(B). These combinations accounted for 62.5% of the resistant strains. Notably, isolates carrying only *mph*(B) (6.5%) fell within the wild-type population, with MICs in the range of 32 to 64 μg/mL (Fig. 1A). Azithromycin showed higher potency on wild-type strains (i.e., MICs of 1 to 16 μg/mL), whereas the potency was lower for tilmicosin (i.e., MICs of 32 to 256 μg/mL), spiramycin (with MICs of 128 to >1,024 μg/mL), and tylosin (with MICs of 256 to >1,024 μg/mL). By using ECOFFinder, tentative ECOFFs separating wild-type and resistant subpopulations for azithromycin, tilmicosin, and spiramycin were set at 8, 256, and 256 μg/mL, respectively (Fig. 1B, C, and E). As observed for erythromycin, the presence of *mph*(B) alone did not increase the MICs of these two macrolides. No tentative ECOFFs for tylosin were determined by ECOFFinder because of the low range (16-fold) of the observed MICs (Fig. 1D).

**TABLE 1:** MICs of erythromycin in 324 clinical E. coli isolates from pigs

MIC (μg/mL)	No. (%) of isolates
≤32	145 (44.8)
64	88 (27.2)
128	16 (4.9)
256	3 (0.9)
512	5 (1.5)
1,024	5 (1.5)
>1,024	62 (19.1)
Total	324 (100)

Genomes were screened for the presence of mutations in the coding sequence of L4 and L22 proteins (*rplD* and *rplV* genes, respectively) in all sequenced strains (*n* = 62). Within the *rplD* gene, a single mismatch (T393C) was identified in 10 isolates, whereas 4 strains carried 2 mismatches in *rplV* (T106C and A147G). However, no correlation with the macrolide susceptibility profile was observed (see Data Set S1 in the supplemental material). In addition, all three mismatches were synonymous mutations.

10.1128/msphere.00402-22.5 DATA SET S1 Antimicrobial resistance and macrolide susceptibility profiles of 62 sequenced Escherichia coli isolates from Danish pigs included in this study. Download Data Set S1, XLSX file, 0.01 MB. Copyright © 2022 Ma et al. 2022 Ma et al. https://creativecommons.org/licenses/by/4.0/ This content is distributed under the terms of the Creative Commons Attribution 4.0 International license.

### Potentiation of different macrolide subclasses by peptidomimetics.

**FIG 2:**
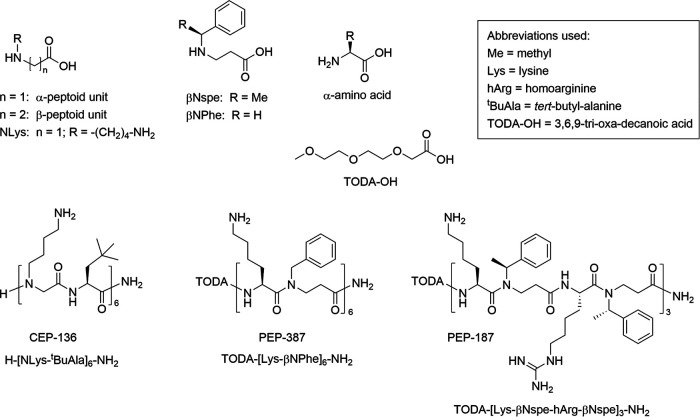
Structures and chemical names for the peptidomimetics investigated as potentiators of macrolides.

Checkerboard analysis was performed to assess peptidomimetic-macrolide synergy in six neomycin-resistant strains representing different genetic lineages and macrolide resistance gene profiles. The three peptidomimetics analyzed in this study (structures and chemical names are shown in Fig. 2) exhibited synergistic interactions (fractional inhibitory concentration index [FICI] < 0.5) with erythromycin (14-membered), azithromycin (15-membered), and tilmicosin (16-membered) in all 6 strains tested (Table 2). The MICs of the three macrolides tested decreased by 4- to 32-fold following exposure to low concentrations of peptidomimetic (0.5 to 8 μg/mL). The magnitude of this decrease was only marginally influenced by strain lineage and macrolide resistance genotype (Table 2). Among the three peptidomimetics tested, PEP-187 displayed slightly stronger macrolide potentiation effects than those seen for PEP-387 and CEP-136. When exposed to PEP-187, the two wild-type strains, KA40 and KB36, had the lowest MICs for azithromycin (0.125 to 0.25 μg/mL), erythromycin (0.5 to 1 μg/mL), and tilmicosin (4 μg/mL), whereas the resistant strains displayed higher MICs (Table 2).

**TABLE 2:** Checkerboard results for the combinations of macrolides (erythromycin, azithromycin, and tilmicosin) and peptidomimetics (PEP-187, PEP-387, and CEP-136) in six clinical E. coli strains

Isolate ID	ST^*a*^	M-R^*b*^ gene(s)	M^*c*^	MIC_A_^*d*^	PEP-187				PEP-387				CEP-136			
					MIC_A(A+B)_^*e*^	MIC_B_^*f*^	MIC_B(A+B)_^*g*^	FICI^*h*^	MIC_A(A+B)_	MIC_B_	MIC_B(A+B)_	FICI	MIC_A(A+B)_	MIC_B_	MIC_B(A+B)_	FICI
115467-0	100	None	ERY	16	1	8	1	0.188	2	16	1	0.188	1	32	1	0.094
			AZI	2	0.25		1	0.25	0.25		1	0.188	0.25		1	0.156
			TIL	32	4		1	0.25	8		0.5	0.281	4		2	0.188
																
116221	88	None	ERY	16	0.5	16	1	0.094	2	32	1	0.156	2	64	1	0.141
			AZI	2	0.125		2	0.188	0.25		1	0.156	0.25		1	0.141
			TIL	64	4		1	0.125	16		1	0.281	8		1	0.141
																
116120-2	88	*erm*(B)	ERY	2,048	128	64	4	0.125	128	128	8	0.125	512	64	4	0.313
			AZI	64	4		1	0.078	8		2	0.141	4		2	0.094
			TIL	1,024	64		4	0.125	128		4	0.156	128		2	0.156
																
117351-6	101	*mph*(A)	ERY	512	16	16	1	0.094	32	32	2	0.125	32	64	2	0.094
			AZI	32	2		0.5	0.094	4		2	0.188	4		1	0.141
			TIL	64	8		0.5	0.156	16		1	0.281	8		1	0.141
																
117039-2	88	*erm*(B), *mph*(A)	ERY	2,048	128	32	1	0.094	256	64	2	0.156	512	64	1	0.266
			AZI	128	8		1	0.094	16		2	0.156	16		2	0.156
			TIL	2,048	128		1	0.094	256		2	0.156	256		1	0.141
																
116244-3	100	*erm*(B), *mph*(A)	ERY	8,192	512	16	1	0.125	1,024	32	4	0.25	512	64	2	0.094
			AZI	1,024	32		1	0.094	64		2	0.125	64		1	0.078
			TIL	2,048	128		2	0.188	256		8	0.375	256		2	0.156

a ST, sequence type.

b M-R, macrolide resistance.

c M, macrolide; ERY, erythromycin; AZI, azithromycin; TIL, tilmicosin.

d MIC_A_, MIC (μg/mL) of macrolide alone.

e MIC_A(A+B)_, MIC (μg/mL) of macrolide in combination with peptidomimetic.

f MIC_B_, MIC (μg/mL) of peptidomimetic alone.

g MIC_B(A+B)_, concentration of peptidomimetic (μg/mL) in each combination leading to the highest synergy.

h FICI is given for the combinations with the highest degree of synergy.

## DISCUSSION

Repurposing of macrolides for treatment of Gram-negative infections has gained interest in the scientific community due to the lack of new effective antimicrobials against Gram-negative bacteria. It has been known for decades that agents capable of increasing outer membrane permeability, such as polymyxin B nonapeptide (at 3 μg/mL) and desacyl polymyxin B (at 1 μg/mL), sensitize wild-type E. coli to azithromycin by factors of 10 and 30, respectively (10). More recently, some studies proved that also low concentrations of peptidomimetics (i.e., at 1 to 8 μM) are sufficient to potentiate the antibacterial activity of azithromycin and clarithromycin by facilitating their penetration across the bacterial envelope, thereby improving access to their intracellular target (14). The present study was conceived to understand whether peptidomimetics could be used as helper drugs to sensitize neomycin-resistant E. coli to macrolides for management of enteric infections in pigs. Such a treatment strategy would be particularly suitable for this veterinary indication in view of the pharmacological properties of 16-membered macrolides, which reach high concentrations in the pig intestinal lumen due to poor absorption (15). Furthermore, several 16-membered macrolides (e.g., tylosin, tilmicosin, and spiramycin) are already licensed for the treatment of pig enteritis caused by other Gram-negative pathogens such as Lawsonia intracellularis and Brachyspira hyodysenteriae. Although the cellular target of macrolides (the 50S ribosomal subunit) is also present in E. coli, these relatively hydrophobic antibiotics are not able to penetrate its highly polar outer membrane. Thus, the only real hurdle to implementing this clinical application in veterinary pig practice is to identify a membrane permeabilizer that is safe, stable, and capable of potentiating veterinary macrolides at concentrations that can be achieved in the pig intestinal tract after oral administration. Here, we provide initial experimental support for this potential veterinary clinical application by showing *in vitro* synergy between veterinary macrolides and three peptidomimetics. These belong to the overall class of peptoid-peptide hybrids, characterized by a high stability toward degradation by pronase and the digestive enzymes trypsin and chymotrypsin (16, 17), as well as low toxicity to mammalian cells (17–19). Moreover, they are readily synthesized chemically by the assembly of dimeric building blocks (18, 20) or tetrameric fragments by solid-phase methods amenable for gram-scale preparation with purification by vacuum liquid chromatography (21).

At submicromolar concentrations, these compounds reduced the MIC of macrolides in wild-type strains to a level that can be achieved in the intestinal tract following oral administration of safe dosages. As the most prominent example, the MIC of tilmicosin in wild-type strains was reduced from 32–64 μg/mL to 4 μg/mL by PEP-187 (Table 2). Approximately 40% of orally dosed tilmicosin is bioactive in pig feces (22). A single oral dose of 10 mg/kg (equivalent to 250 mg tilmicosin/kg feed) resulted in an average concentration of 3.78 μg/mL in the ileum content with a half-life of 7.8 h (23). A concentration above the MIC of 4 μg/mL is therefore likely to be achieved for at least 8 h, on average, if the dose is doubled to 20 mg/kg (500 mg tilmicosin/kg feed) (23). In addition, there can be some accumulation with repeated dosing due to the relatively long half-life, which would result in even more favorable concentrations. Out of the 62 neomycin-resistant isolates, 35 (56.5%) showed a tilmicosin MIC of ≤64 μg/mL (Fig. 1B; also see Data Set 1 in the supplemental material), and thus these are potentially susceptible to combination therapy with PEP-187. These data are promising but require further *in vivo* pharmacological and clinical validation, since the actual concentrations of bioactive drug that can be reached for both tilmicosin and PEP-187 at the ETEC infection site (small intestine) are unknown. Furthermore, other factors may influence the clinical outcome, e.g., the potent immunomodulatory activity of tilmicosin (24) and the effects of health status on drug pharmacokinetics (25).

Notably, our study provides new useful information on the prevalence of acquired macrolide resistance genes in porcine E. coli as well as on how genotypic resistance influences the resistance phenotype in a drug- and gene-specific manner. Overall, acquired genotypic resistance to macrolides was 38.7% in our national collection of clinical E. coli isolated from Danish pig farms in 2020. Certain resistance genes displayed substrate specificity, e.g., *mph*(A) did not increase the MICs of tilmicosin and spiramycin as compared to those of the wild-type strains, while *mph*(B) showed a similar pattern for erythromycin, azithromycin, and tilmicosin (Fig. 1). Based on these results, it appears that the antimicrobial activity of tilmicosin is not affected by phosphorylases encoded by these *mph* genes, further reducing the prevalence of phenotypic resistance to this 16-membered macrolide to 29.0% among neomycin-resistant isolates. Higher proportions of clinical isolates that displayed MICs above those of the wild-type strains were observed for the other two macrolides, for which the MIC distributions could be interpreted by using tentative ECOFFs, namely, erythromycin (32.3%) and azithromycin (32.3%). Altogether, the results on the prevalence of macrolide resistance and gene-drug interactions highlight another positive trait for repurposing tilmicosin against neomycin-resistant E. coli causing pig enteritis in Denmark.

Based on wild-type MIC distributions, azithromycin was confirmed to be the most potent macrolide against E. coli, followed by erythromycin and tilmicosin (Fig. 1). Compared to other macrolides, azithromycin has cationic properties resulting in higher intracellular uptake (9, 26). Furthermore, this new-generation macrolide has a significantly higher oral bioavailability, since it is more resistant to the low pH in the stomach and interacts less extensively with drug transporters and metabolizing enzymes than other macrolides such as erythromycin (27). Due to its superior pharmacodynamic (PD) and pharmacokinetic (PK) properties, azithromycin is by far the most used macrolide in human medicine for the management of a variety of infections, including enteric infections caused by Gram-negative bacteria. Indeed, azithromycin is both clinically and bacteriologically effective in treating human enteric fever caused by Salmonella enterica serovar Typhi or Paratyphi (28). The tentative ECOFF value of azithromycin proposed for porcine E. coli in the present study (i.e., ≤8 μg/mL) is close to that established by EUCAST for closely related *Enterobacterales* such as Salmonella and *Shigella* (≤16 μg/mL). However, azithromycin is better absorbed than tilmicosin, and above all, it is not licensed for veterinary use and is too expensive and important in human medicine to be used in pig production. On the other hand, our results may have implications in human medicine for future treatment of enteric infections caused by *Enterobacterales*. In addition, we set tentative ECOFFs for erythromycin and tilmicosin to 128 and 256 μg/mL, respectively, which are in good agreement with the MIC distribution of wild-type isolates (Fig. 1A and C). The tentative ECOFF for spiramycin was set to 256 μg/mL by ECOFFinder. However, this value is not in line with the presence of known macrolide resistance genes, since four wild-type isolates (6.5%) showed MICs above the ECOFF value (Fig. 1E). Sample size is known to influence ECOFFs (29), and our data set contained only 62 isolates. In addition, E. coli isolates showed a narrow MIC range for spiramycin (from 128 to >1,024 μg/mL), which could have hampered ECOFF analysis.

The biological reasons leading E. coli to acquire macrolide resistance determinants despite intrinsic resistance remain unknown. This phenomenon might be the consequence of exposure to high macrolide concentrations, as these antibiotics are poorly absorbed and tend to accumulate in the intestinal tract of pigs treated orally. Indeed, the presence of *erm*(B) conferred high-level resistance to all the tested macrolides (Fig. 1), and strains possessing both *erm*(B) and *mph*(A) usually had higher MICs of tilmicosin than those having only one of these genes (Fig. 1C), indicating that a combination of different resistance mechanisms is a beneficial strategy for bacteria to resist antibiotics. It was previously reported that the *mph*(A)-encoded phosphotransferase inactivates 14-membered macrolides more efficiently than 16-membered macrolides (29). In agreement with these observations, porcine E. coli strains from our collection carrying *mph*(A) fell outside the wild-type population for erythromycin (14-membered) but were comprised in the wild-type populations for tilmicosin, tylosin, and spiramycin (Fig. 1), which are all 16-membered macrolides. In contrast, the *mph*(B)-encoded phosphotransferase does not exhibit substrate preference (30, 31). Although only a limited number of strains contained this macrolide resistance determinant, we observed that its presence alone only conferred a minimal acquired resistance to any of the macrolides tested (Fig. 1). This might be due to a reduced or incorrect expression of *mph*(B) in E. coli. In a previous study, *mph*(B) was found to confer resistance to macrolides in wild-type E. coli strains, but the authors used a codon-optimized construct of this gene that was expressed using a high-copy-number plasmid (pET28a) (32). In addition to the acquisition of macrolide resistance genes, point mutations at the L4 and L22 ribosomal proteins or 23S rRNA can also be responsible for increased resistance to macrolides according to a previous study (9). Since all the nucleotide substitutions found in the coding sequence of L4 or L22 were synonymous mutations and no correlation with the macrolide susceptibility was observed (Data Set S1), we speculate that the elevated resistance to tylosin and spiramycin in wild-type strains may result from 23S rRNA mutation. Nevertheless, it is unfeasible to screen for mutations in 23S rRNA genes in the present study due to the inherent disadvantage of whole-genome sequencing in assembling rRNA operons (33).

### Conclusion.

This study provides new knowledge on the relationships between macrolide resistance genotypes and phenotypes and highlights a possible innovative strategy for the treatment of E. coli enteritis in pigs. Tilmicosin and the peptidomimetic PEP-187 were shown to be a prospective combination for this potential veterinary application, which deserves further *in vivo* pharmacological investigation in view of the scarcity of effective antibiotics for managing this widespread and economically impacting disease in pig production.

## MATERIALS AND METHODS

### E. coli strain collection.

A total of 441 clinical E. coli from 384 pigs in 324 farms were isolated from January until December 2020 as part of routine diagnostics performed at the National Laboratory for Swine Diseases (SEGES), Kjellerup, Denmark. One isolate per farm was included randomly by using randomization software (https://www.random.org/), leading to 324 isolates. The majority of isolates (95.0%) were derived from small intestinal contents, feces, or liver. Other isolates (5.0%) were from spleen, lung, liver, kidney, joint, navel, peritoneum, bladder, or pleura.

### MIC testing.

Susceptibility to erythromycin was tested in all isolates by using concentrations ranging from 32 to 1,024 μg/mL. A subset of 62 neomycin-resistant isolates were additionally tested for susceptibility to erythromycin using a broader range (8 to 1,024 μg/mL), as well as azithromycin (range, 0.5 to 1,024 μg/mL), tilmicosin (range, 8 to 1,024 μg/mL), tylosin (range, 32 to 1,024 μg/mL), and spiramycin (range, 32 to 1,024 μg/mL). Ethanol was used as solvent for erythromycin, azithromycin, tylosin, and spiramycin, reaching a stock concentration of 102.4 mg/mL, 50 mg/mL, 20 mg/mL, and 20 mg/mL, respectively. Tilmicosin stock solution was prepared in dimethyl sulfoxide (DMSO), and the concentration was 40 mg/mL.

MICs were determined in Mueller-Hinton Broth (MHB) by using the broth microdilution method following the CLSI protocol (34). S. aureus strain ATCC 29213 was used as quality control (QC) strain because the QC range of macrolides for the E. coli QC strain ATCC 25922 is not determined due to the intrinsic resistance of E. coli to macrolides. The QC ranges of erythromycin, azithromycin, tilmicosin, tylosin, and spiramycin for ATCC 29213 were 0.25 to 1 μg/mL, 0.5 to 2 μg/mL, 0.5 to 2 μg/mL, 4 to 8 μg/mL, and 1 to 2 μg/mL, respectively. An epidemiological cutoff (ECOFF) value is the MIC representing the upper limit of a wild-type MIC distribution (35). When possible, tentative ECOFFs were determined for the macrolides tested by using the ECOFFinder datasheet available at https://clsi.org/meetings/susceptibility-testing-subcommittees/ecoffinder/ (accessed on 18 August 2022), and MIC values >1,024 μg/mL were regarded as 2,048 μg/mL. ECOFF values were set to the highest percentage (99.9%) to increase specificity for wild-type isolates and rounded up to the nearest MIC value.

### Genotyping by whole-genome sequencing.

DNA was extracted by using the Maxwell RSC Cultured Cells DNA kit (Promega, WI, USA) following the manufacturer’s instructions. In brief, a single colony of E. coli cells from a blood agar plate was transferred into 5 mL of Luria-Bertani (LB) broth and incubated at 37°C overnight in an orbital shaker. To isolate genomic DNA, 4 μL of RNase was added to 400 μL of the sample culture and kept at room temperature for 10 min. Lysis buffer (100 μL) was added to this mixture, and samples were run on the Maxwell RSC machine (Promega). The quality and quantity of extracted DNA were determined by a NanoDrop 1000 (Thermo Fischer Scientific, MA, USA) and by agarose gel electrophoresis. DNA libraries were constructed by using the Nextera XT library preparation kit (Illumina, CA, USA) following the manufacturer’s protocol, with subsequent sequencing on the MiSeq platform (Illumina).

Raw sequencing reads were assembled by using SPAdes Genome Assembler (v.3.13.1) and quality checked on QUAST (v.5.0.2). Assembled genomes were screened for the presence of resistance determinants by using ABRicate v.1.0.1 (https://github.com/tseemann/abricate) against the ResFinder database (36), and alignment results with identity scores greater than 95% were selected as positive matches. Multilocus sequence typing (MLST) was performed by using MLST 2.0 (Achtman scheme) available at the Center for Genomic Epidemiology (http://www.genomicepidemiology.org). Point mutations in *rplD* and *rplV* genes encoding L4 and L22 50S ribosomal proteins, respectively, were searched in the assembled genomes using reference gene sequences from E. coli MG1655 (GenBank accession no. NC_000913).

### Checkerboard assay.

Macrolide potentiation by peptidomimetics was measured by checkerboard assay in six E. coli strains representative of different sequence types and macrolide resistance gene profiles (Table 2). Three macrolides were tested to represent different subclasses, erythromycin (14-membered), azithromycin (15-membered), and tilmicosin (16-membered). Three peptidomimetics, namely, CEP-136 (14), PEP-187 (13), and PEP-387, were selected as representatives of different subclasses, NLys-based α-peptoid/peptide hybrids, side chain chiral β-peptoid/peptide hybrids with a mixed Lys/hArg content, and achiral β-peptoid/peptide Lys-based hybrids, respectively.

The checkerboard assay was performed as previously described (37) with some modifications. Briefly, the test antibiotic was 2-fold serially diluted along the rows in a 96-well microtiter plate, while the peptidomimetic was 2-fold serially diluted along the columns to create a matrix in which each well contained a combination of both agents at different concentrations. FICI was calculated according to the following formula: FICI = [MIC_A(A+B)_/MIC_A_] + [MIC_B(A+B)_/MIC_B_], where MIC_A(A+B)_ and MIC_B(A+B)_ represent the concentrations of compounds A and B, respectively, in the combination, while MIC_A_ and MIC_B_ represent the MIC of each compound individually. The interaction of the two compounds was interpreted as synergy, antagonism, or indifference for FICI values of ≤0.5, >4.0, and >0.5 to 4.0, respectively (38).

### Solid-phase synthesis and characterization of PEP-387.

The main part of α-peptide/β-peptoid peptidomimetic PEP-387 was assembled on a 9-fluorenylmethoxycarbonyl (Fmoc)-Rink Amide polystyrene resin (loading, 0.7 mmol/g, 0.1 mmol) in a Teflon reactor (10 mL; fitted with a polypropylene filter and a Teflon valve) by standard Fmoc-based solid-phase synthesis using the Fmoc-Lys(Boc)-βNPhe-OH dimeric building block (20). Final Fmoc deprotection was followed by attachment of the N-terminal TODA-OH (5 eq) under conditions identical to those applied for the dimeric building block. Cleavage and simultaneous side chain deprotection gave a crude product that was purified by preparative high-performance liquid chromatography (HPLC) with subsequent lyophilization as previously described (17). For analytical HPLC, retention time (*t*_R_) was 10.19 min (99.7% purity with UV detection at λ of 220 nm). High-resolution matrix-assisted laser desorption ionization–time of flight mass spectrometry (HR-MALDI-TOF MS) was calculated for [M+H]^+^ of 1914.18545 and found 1914.18376, with ΔM = 0.8 ppm. See supplemental material for details on the analytical procedures (Text S1), the structural formula of PEP-387 (Fig. S1), copies of HPLC chromatogram (Fig. S2), and mass spectrum (Fig. S3).

10.1128/msphere.00402-22.1 TEXT S1 General procedures for characterization of PEP-387. Download Text S1, PDF file, 0.3 MB. Copyright © 2022 Ma et al. 2022 Ma et al. https://creativecommons.org/licenses/by/4.0/ This content is distributed under the terms of the Creative Commons Attribution 4.0 International license.

10.1128/msphere.00402-22.2 FIG S1 Structural formula of PEP-387. Download FIG S1, PDF file, 0.01 MB. Copyright © 2022 Ma et al. 2022 Ma et al. https://creativecommons.org/licenses/by/4.0/ This content is distributed under the terms of the Creative Commons Attribution 4.0 International license.

10.1128/msphere.00402-22.3 FIG S2 Analytical HPLC chromatogram for PEP-387. (A) Complete chromatogram. (B) Zoomed chromatogram. Gradient, 0 to 60% B during 10 min. B is 95% MeCN plus 0.1% trifluoroacetic acid (TFA). Retention time (*t*_R_), 10.19 min; purity, 99.7%. Download FIG S2, PDF file, 0.04 MB. Copyright © 2022 Ma et al. 2022 Ma et al. https://creativecommons.org/licenses/by/4.0/ This content is distributed under the terms of the Creative Commons Attribution 4.0 International license.

10.1128/msphere.00402-22.4 FIG S3 HR-MALDI-TOF spectrum for PEP-387. (A) Spectrum calculated for [M+H]^+^ 1914.18545, found 1914.18376; ΔM = 0.8 ppm. (B) The additional peaks seen at higher *m*/*z* represent the [M+Na]^+^ and [M+K]^+^ species as shown on zoomed spectrum. Download FIG S3, PDF file, 0.05 MB. Copyright © 2022 Ma et al. 2022 Ma et al. https://creativecommons.org/licenses/by/4.0/ This content is distributed under the terms of the Creative Commons Attribution 4.0 International license.

### Data availability.

Sequencing data have been submitted to the NCBI Sequence Read Archive (SRA) under BioProject accession no. PRJNA849907.
