# Supplementary material for: Macrolide Resistance and In Vitro Potentiation by Peptidomimetics in Porcine Clinical Escherichia coli
Source: mSphere. 2022 Sep 26;7(5):e00402-22. doi: 10.1128/msphere.00402-22 (PMC9599364; doi:10.1128/msphere.00402-22)
Supplement: TEXT S1 [file msphere.00402-22-s0001.pdf]

General procedures for characterization of PEP-387

Water used for analytical and preparative high-performance liquid chromatography (HPLC) was filtered through a 0.22-µm capsule filter using an Evoqua LaboStar Pro TWF UV system. Purity and retention time of each peptidomimetic were determined by analytical HPLC by using a Phenomenex Luna Omega Polar C18 column (150 × 4.6 mm; particle size: 3 µm; pore size: 100 Å) on a Shimadzu Prominence and Shimadzu Nexera system using an aqueous acetonitrile (MeCN) gradient with 0.1% trifluoroacetic acid (TFA) added (eluent A: 5:95 MeCN–H2O + 0.1% TFA, eluent B: 95:5 MeCN–H2O + 0.1% TFA); a flow rate of 0.8 mL/min was used. For elution of peptidomimetics, a linear gradient of 0% to 60% B during 15 min was used with UV detection at $\lambda = 220$ nm.

High-resolution mass spectrometry (HRMS) spectra were obtained by using a Bruker Solarix XR MS detector.
