# Supplementary figures and images for: Macrolide Resistance and In Vitro Potentiation by Peptidomimetics in Porcine Clinical Escherichia coli
Source: mSphere. 2022 Sep 26;7(5):e00402-22. doi: 10.1128/msphere.00402-22 (PMC9599364; doi:10.1128/msphere.00402-22)

.....

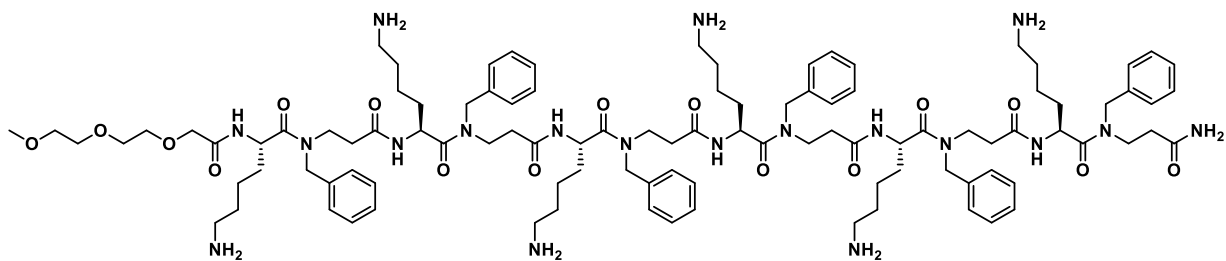

Supplement: FIG S1 [file msphere.00402-22-s0002.pdf]

**A**

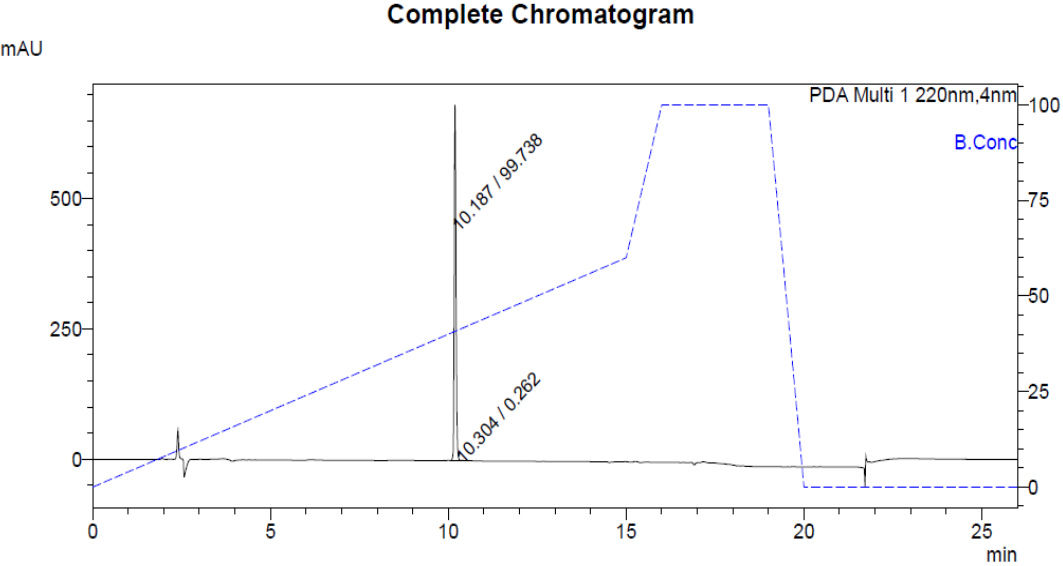

**B**

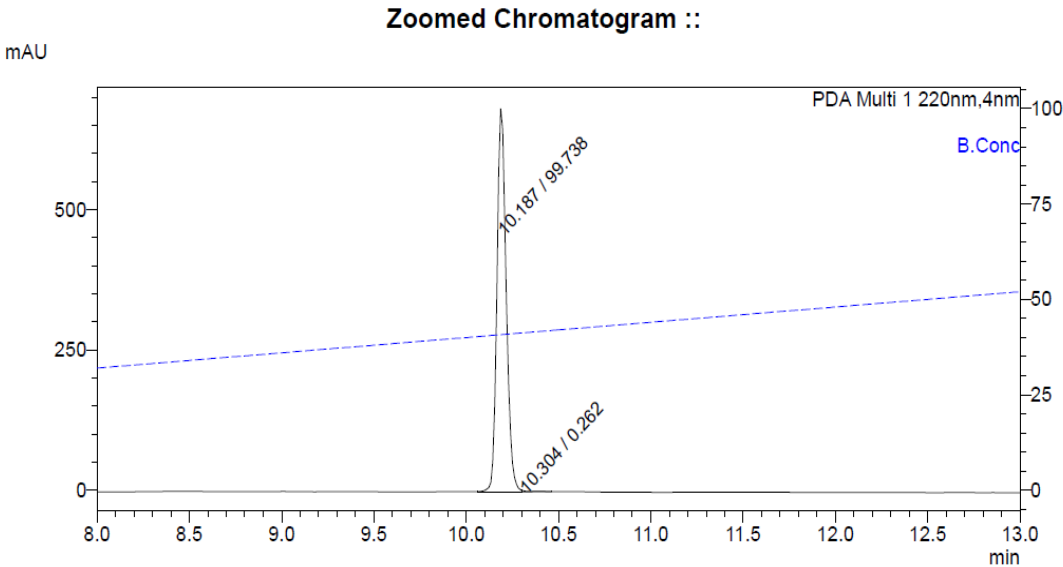

Supplement: FIG S2 [file msphere.00402-22-s0003.pdf]
