# Supplementary material for: Macrolide Resistance and In Vitro Potentiation by Peptidomimetics in Porcine Clinical Escherichia coli
Source: mSphere. 2022 Sep 26;7(5):e00402-22. doi: 10.1128/msphere.00402-22 (PMC9599364; doi:10.1128/msphere.00402-22)
Supplement: FIG S3 [file msphere.00402-22-s0004.pdf]

A

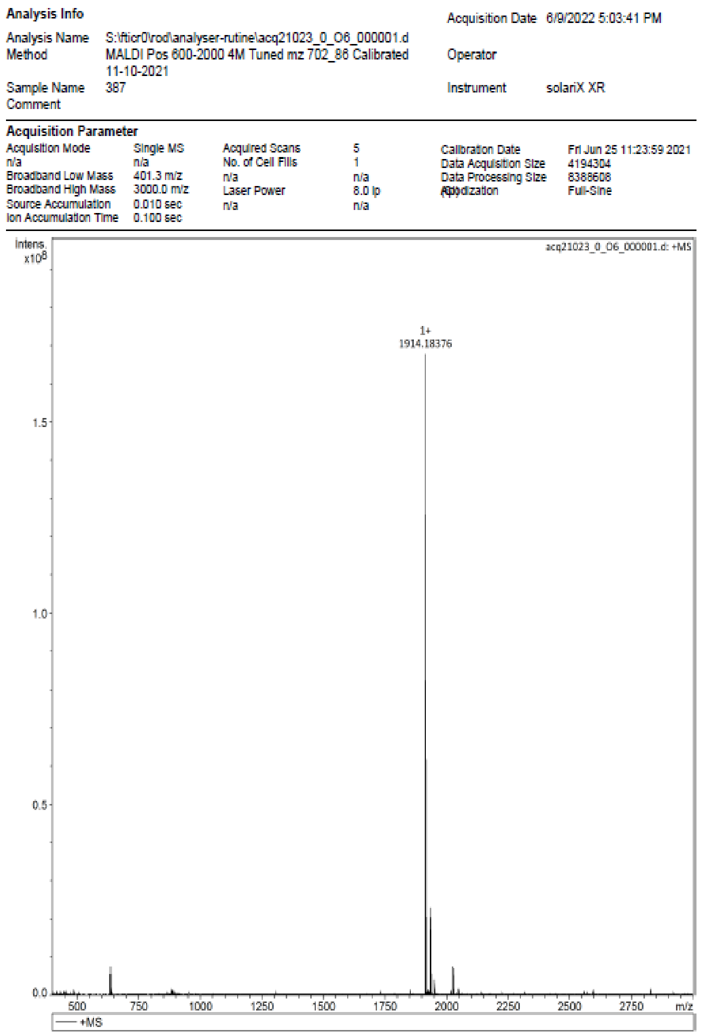
Analysis Info
Analysis Name S:\fticr0\rod\analyser-rutine\acq21023_0_O6_000001.d
Method MALDI Pos 600-2000 4M Tuned mz 702_86 Calibrated 11-10-2021
Sample Name 387
Comment
Acquisition Date 6/9/2022 5:03:41 PM
Operator
Instrument solariX XR
Acquisition Parameter
Acquisition Mode Single MS
n/a n/a
Broadband Low Mass 401.3 m/z
Broadband High Mass 3000.0 m/z
Source Accumulation 0.010 sec
Ion Accumulation Time 0.100 sec
Acquired Scans 5
No. of Cell Fills 1
n/a n/a
Laser Power 8.0 lp
n/a n/a
Calibration Date Fri Jun 25 11:23:59 2021
Data Acquisition Size 4194304
Data Processing Size 8388608
Apodization Full-Sine
Intens. x10^8
acq21023_0_O6_000001.d: +MS
1+
1914.18376
1.5
1.0
0.5
0.0
500
750
1000
1250
1500
1750
2000
2250
2500
2750
m/z
+MS

B

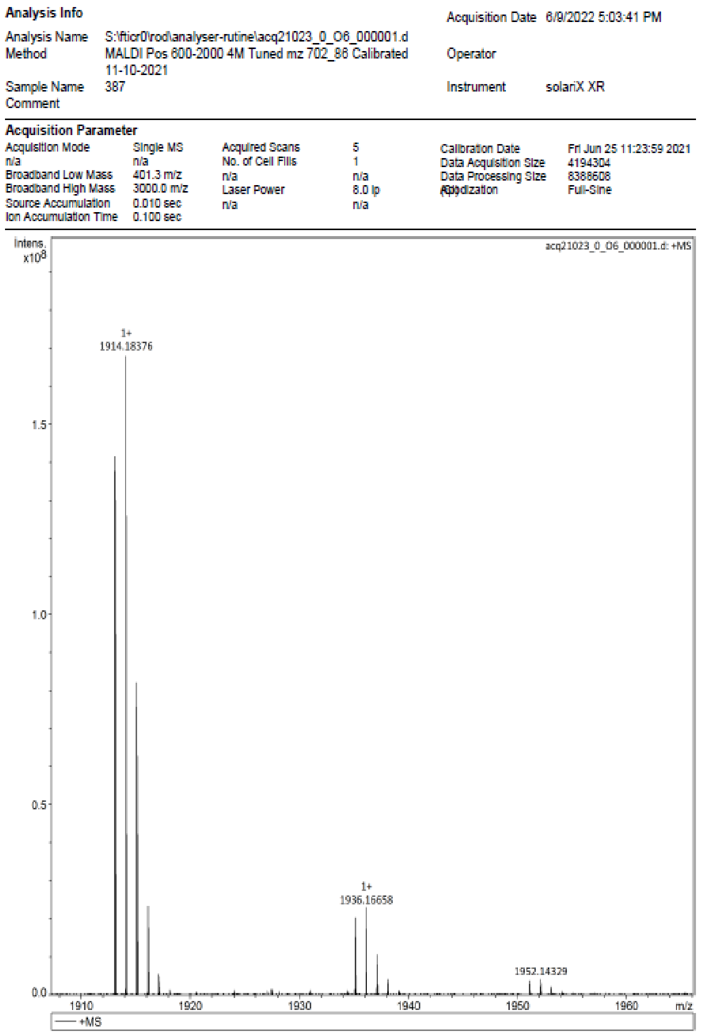
Analysis Info
Analysis Name S:\fticr0\rod\analyser-rutine\acq21023_0_O6_000001.d
Method MALDI Pos 600-2000 4M Tuned mz 702_86 Calibrated 11-10-2021
Sample Name 387
Comment
Acquisition Date 6/9/2022 5:03:41 PM
Operator
Instrument solariX XR
Acquisition Parameter
Acquisition Mode Single MS
n/a n/a
Broadband Low Mass 401.3 m/z
Broadband High Mass 3000.0 m/z
Source Accumulation 0.010 sec
Ion Accumulation Time 0.100 sec
Acquired Scans 5
No. of Cell Fills 1
n/a n/a
Laser Power 8.0 lp
n/a n/a
Calibration Date Fri Jun 25 11:23:59 2021
Data Acquisition Size 4194304
Data Processing Size 8388608
Apodization Full-Sine
Intens. x10^8
acq21023_0_O6_000001.d: +MS
1+
1914.18376
1+
1936.16658
1952.14329
1.5
1.0
0.5
0.0
1910
1920
1930
1940
1950
1960
m/z
+MS
